# Unravelling delayed therapy escape after thalamic deep brain stimulation for essential tremor? – Additional clinical and neuroimaging evidence

**DOI:** 10.1016/j.nicl.2022.103150

**Published:** 2022-08-11

**Authors:** Bastian E.A. Sajonz, Marvin L. Frommer, Isabelle D. Walz, Marco Reisert, Christoph Maurer, Michel Rijntjes, Tobias Piroth, Nils Schröter, Carolin Jenkner, Peter C. Reinacher, Joachim Brumberg, Philipp T. Meyer, Ganna Blazhenets, Volker A. Coenen

**Affiliations:** a Department of Stereotactic and Functional Neurosurgery, Medical Center – University of Freiburg, Faculty of Medicine, University of Freiburg, Freiburg im Breisgau, Germany; b Department of Neurology, Medical Center – University of Freiburg, Faculty of Medicine, University of Freiburg, Freiburg im Breisgau, Germany; c Department of Sport and Sport Science, University of Freiburg, Freiburg im Breisgau, Germany; d Department of Neurology, Kantonsspital Aarau, Aarau, Switzerland; e Clinical Trials Unit, Medical Center – University of Freiburg, Faculty of Medicine, University of Freiburg, Freiburg im Breisgau, Germany; f Fraunhofer Institute for Laser Technology (ILT), Aachen, Germany; g Department of Nuclear Medicine, Medical Center – University of Freiburg, Faculty of Medicine, University of Freiburg, Freiburg im Breisgau, Germany; h Center for Deep Brain Stimulation, University of Freiburg, Germany; i Center for Basics in Neuromodulation (Neuromod Basics), Faculty of Medicine, University of Freiburg, Freiburg im Breisgau, Germany

**Keywords:** ACPC, intercommissural, DBS, deep brain stimulation, DRT, dentatorubrothalamic tract, FDG, Fluorodeoxyglucose, FTMTRS, Fahn-Tolosa-Marin Tremor Rating Scale, PC, posterior commissure, PET, Positron Emission Tomography, rCBF, regional cerebral blood flow, SARA, Scale for the Assessment and Rating of Ataxia, TCPP, total charge per pulse, TEED, total electrical energy delivered, TUG, Timed Up and Go, Deep brain stimulation, Essential tremor, Habituation, Delayed therapy escape, PET

## Abstract

**Background:**

Delayed therapy escape after thalamic deep brain stimulation (DBS) for essential tremor is a serious yet frequent condition. It is often difficult to detect this process at onset due to its gradual evolution.

**Objective:**

Here we aim to identify clinical and neuroimaging hallmarks of delayed therapy escape.

**Methods:**

We retrospectively studied operationalized and quantitative analyses of tremor and gait, as well as [^18^F]fluorodeoxyglucose (FDG) PET of 12 patients affected by therapy escape. All examinations were carried out with activated DBS (ON) and 72 h after deactivation (OFF_72h_); gait and tremor were also analyzed directly after deactivation (OFF_0h_). Changes of normalized glucose metabolism between stimulation conditions were assessed using within-subject analysis of variance and statistical parametric mapping. Additionally, a comparison to the [^18^F]FDG PET of an age-matched control group was performed. Exploratory correlation analyses were conducted with operationalized and parametric clinical data.

**Results:**

Of the immediately accessible parametric tremor data (i.e. ON or OFF_0h_) only the rebound (i.e. OFF_0h_) frequency of postural tremor showed possible correlations with signs of ataxia at ON. Regional glucose metabolism was significantly increased bilaterally in the thalamus and dentate nucleus in ON compared to OFF_72h_. No differences in regional glucose metabolism were found in patients in ON and OFF_72h_ compared with the healthy controls.

**Conclusions:**

Rebound frequency of postural tremor seems to be a good diagnostic marker for delayed therapy escape. Regional glucose metabolism suggests that this phenomenon may be associated with increased metabolic activity in the thalamus and dentate nucleus possibly due to antidromic stimulation effects. We see reasons to interpret the delayed therapy escape phenomenon as being related to long term and chronic DBS.

## Highlights

• Rebound tremor frequency seems to be a good diagnostic marker for delayed therapy escape.
• Ataxia seems to play a major role in delayed therapy escape.
• Therapy escape may be due to increased neuronal activity in the tremor network.

## 1 Introduction

Essential Tremor is one of the most common movement disorders in adulthood. It shows an age-related increase in prevalence (Louis and Ferreira, 2010, Louis et al., 1998) and causes disability and social withdrawal (Koller et al., 1986). Deep brain stimulation (DBS) is a safe and effective treatment in pharmacotherapy resistant essential tremor (Chopra et al., 2013). While the classical target point for DBS in essential tremor is the ventral intermediate nucleus of the thalamus (Benabid et al., 1996), there is increasing evidence that the stimulation must address the dentatorubrothalamic tract (DRT) (Akram et al., 2018, Coenen et al., 2020, Coenen et al., 2014, Coenen et al., 2011a, Coenen et al., 2011b, Fenoy and Schiess, 2017, Sammartino et al., 2016). However, after initial improvement of tremor following DBS surgery up to 40 % of the patients experience a loss of efficacy that has been referred to in the literature as *habituation, tolerance and late failure,* respectively (Chiu et al., 2020, Pilitsis et al., 2008). This unfortunate condition cancels out the benefits of the therapy despite repeated stimulation adjustments. Several studies linked it to disease-related (progression, preexisting cerebellar dysfunction, coexisting demyelinating neuropathy), stimulation-related (pulse width, tolerance, antidromic effects on adjacent cerebellothalamic fibers) and surgery-related aspects (electrode location, shorter disease duration at surgery, older age at surgery) (Anthofer et al., 2017, Chiu et al., 2020, Favilla et al., 2012, Merchant et al., 2018, Patel et al., 2014, Pilitsis et al., 2008, Reich et al., 2016, Shih et al., 2013).

The phenomenon was also noticed in observational studies focussing on outcomes after DBS in essential tremor (Benabid et al., 1996, Hariz et al., 1999, Koller et al., 2001, Papavassiliou et al., 2004, Sydow et al., 2003, Zhang et al., 2010). In general, comparability of studies is limited due to their focus on selected aspects of the phenomenon, which moreover lacks a consensus definition (Fasano and Helmich, 2019; cf. Patel et al., 2014, Peters and Tisch, 2021). Fasano and Helmich (2019) defined this phenomenon as “therapy escape” and coined a consistent nomenclature of associated processes. Regarding the aspect of recurrent tremor over time (but not ataxia) the data by Paschen et al. (2019) suggest that worsening can be attributed to disease progression (87 %) rather than habituation to stimulation (13 %). However, the following strategies provided clinical alleviation and focused on delayed progressive ataxia and progressive (usually intentional) tremor, suggesting a stimulation-induced genesis: (1) optimization of stimulation parameters (Contarino et al., 2017), especially employing pulse width reduction (Kroneberg et al., 2019, Soh et al., 2019); (2) pausing stimulation (Garcia Ruiz et al., 2001, Reich et al., 2016); and (3) surgery with revision (Coenen et al., 2017) or implant of an additional DBS electrode (Isaacs et al., 2018, Yu et al., 2009).

Overall, strategies to treat patients with delayed therapy escape remain laborious and scarce. Due to its gradual evolution it is also difficult to detect and monitor changes in the usual outpatient setting.

In this retrospective study we analyzed the data gathered in a diagnostic work up of 12 patients from our center who were severely affected by delayed therapy escape, before optimization of their stimulation parameters. We sought to identify potential hallmarks of therapy escape by exploratory analysis of directly accessible tremor features. In addition, we used positron emission tomography (PET) with [^18^F]fluorodeoxyglucose (^18^FDG), to assess regional cerebral glucose metabolism, an established biomarker of neuronal function (Kennedy et al., 1975), during stimulation conditions (ON and OFF_72h_). We hypothesized to find effects within the tremor network.

## 2 Methods

### 2.1 Patients and examination schedule

The present retrospective study complies with the declaration of Helsinki and was approved by the local ethics committee. All patients gave their written informed consent prior to participating in the study. In this retrospective analysis we included patients with bilateral thalamic DBS for essential tremor, if the following was true: (1) patients reported good initial tremor improvement after DBS implantation, but complained of recurrent disabling tremor beginning 12 months after operation or later despite several stimulation changes in the outpatient setting. (2) patients had received a clinical work up including PET imaging similar as previously described (Reich et al., 2016). Six of these patients had received their DBS implantation at another institution. A clinical test battery for tremor and ataxia, a quantitative tremor analysis, and a vision-based motion capture were conducted with the chronically used stimulation setting (ON), with switched off stimulation (OFF_0h_) and after a 72 h wash-out phase with switched off stimulation (OFF_72h_). Measurements in OFF_0h_ were obtained directly after the corresponding ON measurements. When available, preoperative values (PreOP) for postural tremor frequency and FTMRS were gathered. In addition, we determined the timespan of subjectively perceived satisfactory DBS treatment after implantation and stimulation initiation by interviewing patients and reviewing medical records when available. To give an impression of the further course after stimulation adjustment with pulse width reduction we also included supplementary follow up data (rebound tremor frequency, SARA).

### 2.2 Outcomes

#### 2.2.1 Clinical test battery

The Fahn-Tolosa-Marin Tremor Rating Scale (FTMTRS) (Fahn et al., 1993) and the Scale for the Assessment and Rating of Ataxia (SARA) (Schmitz-Hübsch et al., 2006) were used. The examination was recorded on video in the conditions mentioned above. Several months after discharge of the last patient, the videos of all patients were independently presented to both initial examiners in a randomized fashion (across patients and stimulation settings) and the mean values of both examiners were used for further analysis. In addition to the overall SARA score we calculated a modified SARA score without item 6 (nose-finger test measuring tremor) like Roque et al. (2021). SARA values of the first patient are missing for procedural reasons. Clinical examinations additionally comprised malleolar assessment of pallesthesia as a marker of possible polyneuropathy with a Rydel-Seiffer tuning fork (scale 0–8) in all but one patient.

#### 2.2.2 Quantitative tremor analysis

Patients sat comfortably with their forearms on armrests extending their hands and fingers horizontally against gravity. We recorded postural tremor of both hands in the conditions mentioned above with accelerometry and non-invasive electromyography using a custom-made device with software by Lauk et al. (1999). Fourier analysis was used to determine the tremor frequency. The total power of postural tremor was measured in milli-gravities^2^/µV^2^.

PreOP values of postural tremor frequency are missing in two patients.

#### 2.2.3 Vision-based motion capture

Quantitative analysis of gait in a timed-up-and-go task (TUG) was performed with the marker-less vision-based motion capture system TheCaptury (The Captury GmbH, Saarbrücken, Germany) processing the data of 12 RGB cameras running at 100 Hz in a room measuring approximately 28 m^2^. The mean step length of three runs per above mentioned condition was used for further statistical analysis.

#### 2.2.4 Stimulation Parameters and Visualization of Active Contacts

Stimulation parameters were obtained from all patients and the total electrical energy delivered (TEED) was calculated according to Koss et al. (2005) as well as the total charge per pulse (TCPP). Stereotactic intercomissural (ACPC) coordinates of active (cathodal) electrode contacts were determined after coregistration of individual MRI and CT data with Brainlab Elements (Brainlab AG, Munich, Germany). In cases with more than one active contact (including interleaved programs) the value of the resulting center along the electrode was used. For visualization of the patients’ active contacts with regard to the Vim a normalization to MNI space (MNI 2009b *asym*.) was conducted by a joint registration of the T1- and T2-weighted contrast using the ANTS normalization toolbox (Avants et al., 2011). The Vim segmentation was obtained from Ewert et al. (2018). Lead localization using CT data was performed using an in-house MATLAB code followed by manual verification. For visualization the NORA medical imaging platform was used (https://www.nora-imaging.org).

#### 2.2.5 [^18^F]FDG PET imaging

All PET scans were acquired on a fully digital Vereos PET/CT scanner (Philips Healthcare, The Netherlands). After the subjects fasted for at least 6 h, they were intravenously injected with 214 ± 9 MBq (at ON) and 215 ± 9 MBq (at OFF_72h_) [^18^F]FDG under normoglycemic, resting conditions (eyes open and ears unplugged at ambient noise). A 10-min PET scan was acquired starting 50 min after injection, during which the position of the patient’s head was gently restrained with an elastic tape and carefully monitored. Using low-dose CT for attenuation correction, a fully corrected emission dataset was reconstructed with the vendor-specific, line-of-response time-of-flight ordered-subsets 3-dimensional iterative reconstruction algorithm using spherically symmetric basis functions (number of iterations, 5; number of subsets, 11; 2-mm Gaussian post-filtering; resulting voxel size, 1.0 × 1.0 × 1.0 mm), yielding a reconstructed, isotropic image resolution of approximately 4.5–5 mm full width at half maximum (FWHM).

All processing steps were implemented with an in-house pipeline in MATLAB (The MathWorks, Inc., Natick, Massachusetts, United States) and Statistical Parametric Mapping 12 (SPM) software (https://www.fil.ion.ac.uk/spm). [^18^F]FDG PET scans were spatially normalized to an in-house [^18^F]FDG PET template in Montreal Neurologic Institute space (Collins et al., 1994). After proportional scaling of individual voxel-wise [^18^F]FDG uptake to brain parenchyma (from SPM tissue probability map with probability for both gray and white matter of at least 50 %), data were smoothed with an isotropic Gaussian kernel of 8 mm FWHM. PET data of one patient were flipped (left to right) to match the most affected side of the rest of the patients. Whole-brain voxel-based changes of normalized glucose metabolism as a marker of regional neuronal activity between the ON and OFF_72h_ conditions were assessed using one-way within-subject ANOVA calculated with SPM. Results were thresholded at a false discovery rate (FDR)-corrected *P* value of 0.05 (extent threshold > 150 voxels) and separate clusters were converted to binary regions of interest (ROI) for ROI-based analysis. Anatomical positions of defined clusters were assigned based on the cluster’s peak-level coordinates employing AAL2 (Rolls et al., 2015) or SUIT atlas (Diedrichsen et al., 2011, Diedrichsen et al., 2009). Mean normalized [^18^F]FDG uptake was calculated for each ROI unilaterally, with any bilateral cluster being split at the midline (x = 0) for further correlation analyses.

Thirteen age- and sex-matched healthy control subjects had a PET acquired with the same methods (recruited by local advertisement) and served as control cohort. They were healthy according to medical history (no neurologic or psychiatric condition or any other relevant comorbidity) and unimpaired in a neuropsychological evaluation, had no neurological deficit on clinical examination and normal MRI findings of the brain. Whole-brain voxel-based comparison between patients and controls was performed for each stimulation condition separately using a two-sample *t* test in SPM (FDR-corrected *P* < 0.05, extent threshold > 150 voxels).

### 2.3 Statistical analysis

Statistical analysis was performed with SPSS (version 25, IBM, Armonk, NY, USA) and R 4.1.0 (https://www.R-project.org/) to exploratorily assess the following associations using Pearson’s product-moment correlation coefficient: (1) associations between easily obtainable quantitative tremor features (i.e. in ON and OFF_0h_) and signs of ataxia (SARA-Score, step length) to identify potential hallmarks of delayed therapy escape, (2) associations between pallesthesia and the course of ataxic symptoms from ON to OFF_72h_, and (3) associations between metabolism in the clusters (ROI-based [unilaterally], both ON and OFF_72h_) and signs of ataxia (SARA-Score, step length), stimulation parameters (therapeutic current, TEED, TCPP) and frequency of postural tremor at the most affected side. Cohen’s d was calculated to assess the magnitude of effect size between glucose metabolism at different stimulation conditions. Results of exploratory analyses are reported without p-values and interpreted descriptively. To estimate the effects of age, sex, disease duration and time elapsed since DBS implantation on the main outcomes multiple linear regression models were calculated.

### 2.4 Data availability

All data are available upon reasonable request and approval of the local ethics committee from the corresponding author.

## 3 Results

All twelve consecutive patients who had received a clinical work up for delayed therapy escape as described above were included in the analyses. Demographic and clinical characteristics are provided in Table 1.

**Table 1:** Demographic and basic clinical data.

	data distribution
	*n*
Sex (Male:Female)	9:3
Handedness (Right:Left)	12:0
Most affected side (i.e. tremor and ataxia) as indicated by the patient at time of PET (Right:Left)	1:11
	mean ± SD (range)
Age (years)	70 ± 7 (58–80)
Disease duration (years)	39 ± 16 (14–66)
Time since DBS implantation (years)	6.4 ± 4.6 (1.1–12.7)
	median ± IQR (range)
Time patients were satisfied with DBS treatment (months)^1^	21 ± 31 (13–144)

1 Missing data in one patient.

### 3.1 Evaluation of Tremor and Ataxia

**Fig. 1:**
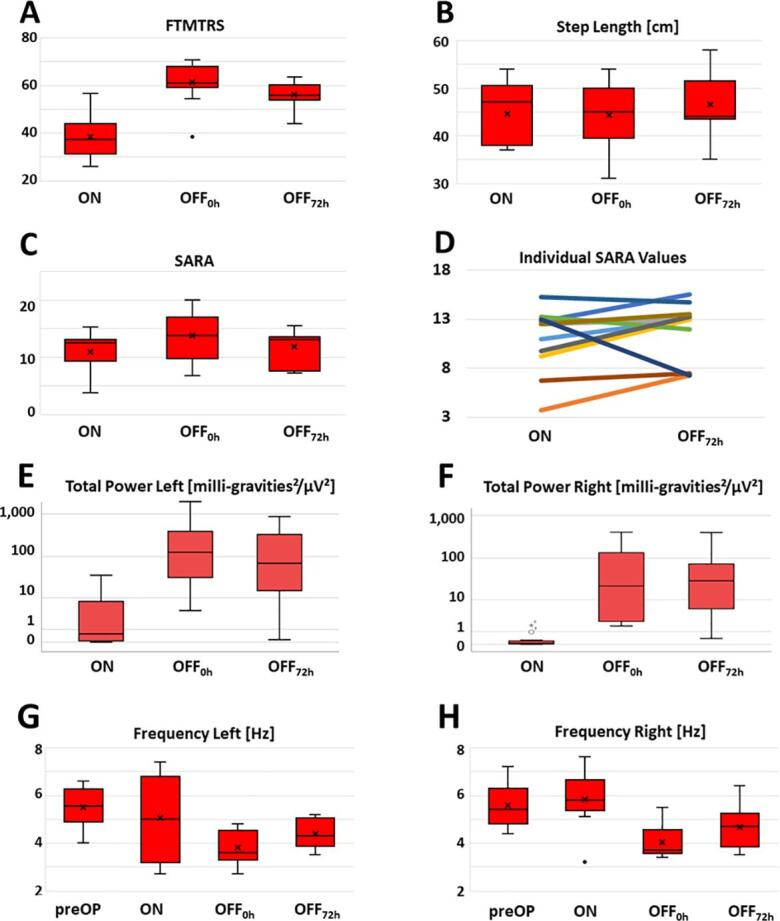
Distribution of tremor and ataxia parameters across stimulation conditions: (A) Fahn-Tolosa-Marin Tremor Rating Scale (FTMTRS); (B) Step Length derived from vision-based motion capture; (C) Scale for the assessment and rating of ataxia (SARA); (D) individual SARA values at ON and OFF_72h_; (E) total power of left postural tremor, (F) total power of right postural tremor, (G) frequency of left postural tremor, (H) frequency of right postural tremor. Missing values: SARA in one patient, step length missing for one patient both in the OFF_0h_ and OFF_72h_ condition and two other patients in either the OFF_0h_ or OFF_72h_ condition. (For interpretation of the references to colour in this figure legend, the reader is referred to the web version of this article.)

Generally, the patients were severely affected by tremor. Considering SARA item 6 only 2 patients showed action tremor with an amplitude ≤ 2 cm on the right hand in the ON condition. Tremor worsened in the OFF_0h_ condition (rebound, tremor increase). After recovery from rebound (OFF_72h_) tremor was still increased as compared to ON condition suggesting a tremor alleviating effect of the stimulation per se (Fig. 1 A, E-F). Regarding signs of ataxia (SARA/step length) we observed no effect across the conditions in the patient group (Fig. 1 B-C).

Comparing FTMTRS (Fig. 1) and the total power of postural tremor (Fig. 1 A, E-F) we find the rebound phenomenon at OFF_0h_ confirmed. The frequency of postural tremor showed a differential course across stimulation conditions: Switching off stimulation resulted in a frequency drop compared to preoperative and ON conditions. The mean frequency of rebound tremor (OFF_0h_) was below 4 Hz for both hands. Within the 72 h wash out phase only a partial recovery occurred (Fig. 1 G-H). FTMTRS values from preOP were available in 3 patients. All of them showed an improvement 12 months postoperatively and then a deterioration towards the baseline of this study (ON) (Supplementary Fig. S1).

**Fig. 2:**
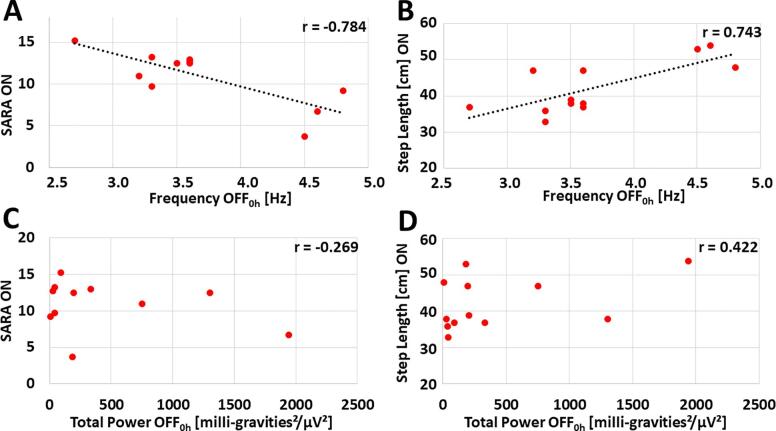
Correlation analyses in search of a directly accessible (i.e. in ON or OFF_0h_) marker of postural tremor of the most affected side for signs of ataxia in ON. Correlation of (A) SARA ON with frequency OFF_0h_, (B) step length at ON with frequency OFF_0h_, (C) SARA ON with total power OFF_0h_ and (D) step length ON with total power OFF_0h_. Missing values for SARA in 1 patient. Regression lines are depicted for correlations with large effect size (exceeding |r| ≥ 0.5). (For interpretation of the references to colour in this figure legend, the reader is referred to the web version of this article.)

Exploratory analyses of the data showed possible correlations of postural tremor rebound frequency (OFF_0h_) with signs of ataxia at ON (Fig. 2): Low rebound tremor frequencies of the most affected side - but not total power of rebound postural tremor (OFF_0h_) - were associated with higher SARA values at ON (*r* = -0.784) and smaller step length at ON (*r* = 0.743). To explore whether these findings are exclusively driven by rebound tremor, we repeated the analysis after excluding item 6 (nose-finger test measuring tremor) of SARA. This measure did not affect the general results (frequency: *r* = -0.776; total power: *r* = -0.364). Further exploratory correlation analyses with other directly accessible (i.e. ON and OFF_0h_) quantitative features of postural tremor (in particular, frequency ON, difference in frequency ON-OFF_0h_, frequency preOP, total power ON) did not show any correlations with large effect size (i.e. exceeding |r| ≥ 0.5, Supplementary Fig. S2). We found the same pattern with signs of ataxia at OFF_0h_ (i.e. SARA and step length, Supplementary Fig. S3). Of note, malleolar pallhypesthesia as a very sensitive marker of polyneuropathy was a common phenomenon in our patients (mean of left and right malleolus ± SD: 3.8 ± 1.9). This parameter showed possible associations with SARA values at OFF_0h_ and OFF_72h_ while associations with SARA values at ON only had a small to medium effect size (Supplementary Fig. S4).

In three patients ataxia was severely exacerbated with switched-off stimulation, so they were not able to safely attend the vision-based motion capture of TUG at the following instances: one patient both in the OFF_0h_ and OFF_72h_ condition, one patient in the OFF_0h_ condition only and another patient in the OFF_72h_ condition only.

In multiple linear regression models the factors age, sex, disease duration and time elapsed since DBS implantation did not have an effect on the association of SARA score or step length with rebound tremor frequency.

The further clinical course following the OFF_72h_ examination after stimulation adjustment involving pulse width reduction is shown in Supplementary Fig. S5. Follow up data was obtained 24 ± 10 months later (mean ± SD). Five patients showed an improvement of their SARA ON values and their rebound tremor tended to increase in frequency.

### 3.2 Evaluation of Regional Cerebral Metabolism

**Fig. 3:**
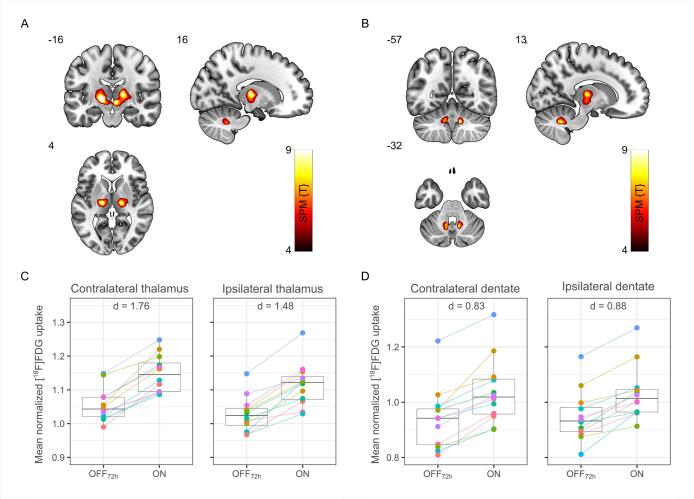
Results of whole-brain voxel-wise analysis of regional cerebral glucose metabolism during stimulation (ON) compared to 72 h wash out phase (OFF_72h_) with Statistical Parametric Mapping (SPM). Voxels of significant increase (false discovery rate corrected, P < 0.05) in normalized regional [^18^F]FDG uptake are located in thalamus (A) and dentate nuclei (B). Corresponding box plots (grey) and individual values of mean normalized [^18^F]FDG uptake (colored) show results of the ROI-based analysis: activation of thalamus (C) and dentate (D) for both contralateral and ipsilateral sides (with regard to the most affected side indicated by the patient at the time of the PET) at ON compared to OFF_72h_ condition. SPM {T} values are color coded and overlaid onto an MRI template. Images are presented in neurologic orientation, i.e., left corresponds to the patients' left body side; numbers denote corresponding position in mm. Cohen’s d values report the effect size. (For interpretation of the references to colour in this figure legend, the reader is referred to the web version of this article.)

**Fig. 4:**
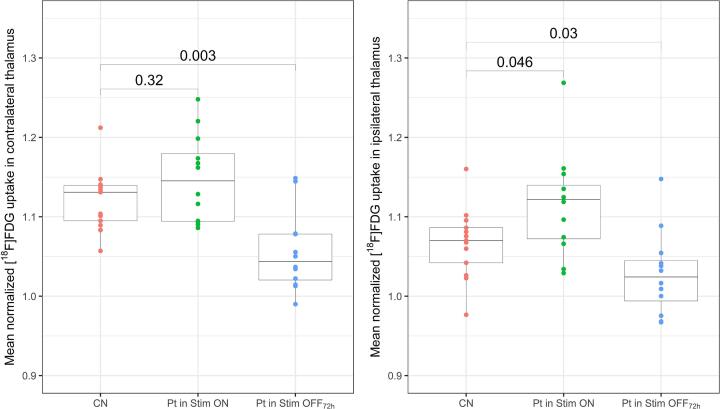
Results of ROI-based analysis of glucose metabolism in thalamus in controls (resting state), patients during stimulation (ON) and 72 h wash out phase (OFF_72h_). Box plots (grey) and individual values of mean normalized [^18^F]FDG uptake (colored) show significant differences in uptake in thalamus for patients (Pt) compared to controls (CN) for both stimulation conditions at contralateral and ipsilateral sides (with regard to the most affected side indicated by the patient at the time of the PET). For controls, right and left anatomical sides were evaluated, respectively. Numeric values report significance of pairwise comparison. (For interpretation of the references to colour in this figure legend, the reader is referred to the web version of this article.)

Whole-brain voxel-wise ANOVA revealed significantly increased metabolism of the thalamus and dentate nucleus bilaterally at ON compared to OFF_72h_ (FDR-corrected *p* < 0.05, height threshold T = 4.64, Fig. 3 A-B). Both thalamic clusters extend into the midbrain. The individual patterns of metabolic activity across the reported anatomical regions are displayed in Fig. 3 C-D. Multiple linear regressions did not find any associations of sex or time related factors (age, disease duration, time elapsed since DBS implantation) with this pattern. Results of the ROI-based analysis indicated that during thalamic stimulation, metabolism of contralateral thalamus (i.e. contralateral to the most affected side indicated by the patient at the time of the PET) correlated positively with the metabolism of dentate nucleus (*r* = 0.58 and *r* = 0.61 for contralateral and ipsilateral dentate, respectively). Moreover, there was an association between metabolism of thalamus at ON and frequency of rebound tremor (OFF_0h_): Patients with a higher [^18^F]FDG uptake in the thalamus at ON tend to have lower frequencies of rebound tremor on the most affected side at OFF_0h_ (Supplementary Fig. S6). Further exploratory analyses with signs of ataxia (SARA-Score, step length) and stimulation parameters (therapeutic current, TEED, TCPP) did not yield any correlation with a large effect size (all |*r*| < 0.5). In whole-brain voxel-wise analysis no differences in glucose metabolism were found in patients compared to healthy controls for either of the stimulation conditions. ROI-based analysis of the thalamus showed that average thalamic metabolism of controls is ranging between Stim ON and OFF_72h_ conditions (Fig. 4).

### 3.3 Electrode positions

**Fig. 5:**
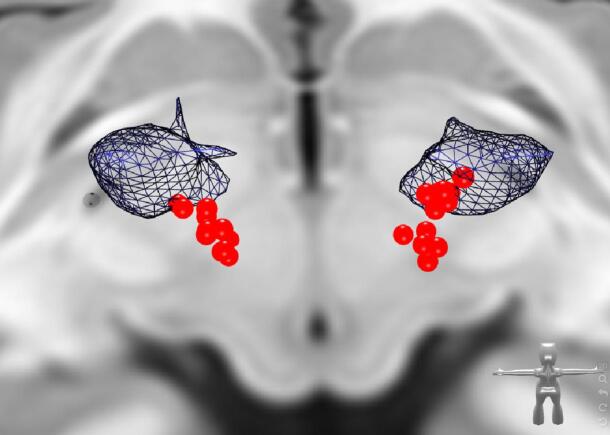
Distribution of electrode contacts in MNI space. Electrode contacts (red spheres) derived from patient imaging to demonstrate their distribution in the posterior third of the Vim (blue) extending into the caudal Zona incerta as conventionally done. View from superoposterior. (For interpretation of the references to color in this figure legend, the reader is referred to the web version of this article.)

The distribution of active electrode contacts is illustrated in Fig. 5. Supplementary Tables S1 and S2 show individual electrode contact positions during chronic stimulation and for intercommissural (ACPC) plane penetration. Supplementary Table S3 gives stimulation parameters.

## 4 Discussion

By combining a detailed clinical workup with state-of-the-art [^18^F]FDG PET imaging, contrasting ON and OFF_72h_ conditions, we were able to identify distinctive clinical and imaging patterns, which allow us to draw further conclusions about the delayed therapy escape after thalamic stimulation for essential tremor.

In our sample of affected patients we found evidence that this phenomenon may be diagnosed by detection of a low rebound (OFF_0h_) frequency of postural tremor and is accompanied by bilaterally elevated metabolic activity in the thalamus and dentate nucleus.

### 4.1 Low Rebound Tremor Frequency as a Marker for Therapy Escape

Frequency is used for classification of tremor syndromes among other tremor characteristics (Bhatia et al., 2018). In particular, low frequencies below 5 Hz are associated with intention tremor syndromes and Holmes Tremor (Bhatia et al., 2018), while essential tremor typically has a higher frequency and values below 4 Hz do not occur (Deuschl et al., 1996). Although one of the patients showed low-frequency (3.1 Hz) resting tremor on the left at ON during the tremor analysis with hands hanging down from the arm rest (data not shown), this tremor always ceased with complete relaxation (e.g. patient lying down flat). Three other patients showed the same phenomenon at least in one hand at OFF_0h_. None of these patients had signs of bradykinesia or rigidity. Thus, from a clinical perspective the rebound tremor can be classified as an intention tremor syndrome (combined with other signs of ataxia) superimposed on essential tremor. Basically, the underlying process leading to a delayed therapy escape seems to be ataxia, because the correlations of rebound tremor frequency and SARA remained stable irrespective of the fact whether the tremor item was included in the SARA score. This observed rebound intention tremor syndrome with ataxia suggests a (temporary) lesion of the cerebellothalamic pathway/DRT (Bhatia et al., 2018, Bodranghien et al., 2016). Thalamic DBS in essential tremor has a similar outcome to (sub)thalamotomy and can be regarded as a temporal disruption of abnormal neuronal activity (Chiken and Nambu, 2016). Thalamic stimulation affects cerebellar symptoms in two ways: improvement can be achieved at common therapeutic amplitudes (Fasano et al., 2010, Herzog et al., 2007, Roque et al., 2021), whereas supratherapeutic stimulation can instantaneously elicit symptoms of ataxia during the applied time (Fasano et al., 2010, Groppa et al., 2014, Hidding et al., 2019) although not instantaneously exacerbating tremor or changing its phenotype. Measures to widen the therapeutic window can reduce ataxia as an immediate side effect of stimulation - e.g. pulse width reduction (Choe et al., 2018, Moldovan et al., 2018) and directional stimulation (Roque et al., 2021). It remains unclear whether ataxia as an immediate side effect of (supratherapeutic) stimulation is a prerequisite for the delayed therapy escape, and whether the aforementioned measures can prevent it, if applied at initial programming. Despite similarities, delayed therapy escape clearly differs from ataxia due to abrupt supratherapeutic stimulation: The clinical phenotype of abrupt supratherapeutic stimulation is comparable to a sudden lesion of the DRT (Boutet et al., 2018, Marek et al., 2015) while delayed therapy escape evolves gradually and deactivation of stimulation leads to rebound of intention tremor and ataxia, which impedes immediate relief by measures that have been shown to alleviate ataxia in cases without delayed therapy escape. This rebound exacerbation can be terminated promptly by switching the stimulation back on (even at chronically high amplitudes), bringing the patient back to the baseline level of symptoms (ON). The rebound effect on tremor has been shown to reach a plateau 30–60 min after switching off the stimulation (Paschen et al., 2019), whereas the rebound in ataxia seems to be modulated in a different way and time frame. While Reich et al. (2016) observed a complete recovery at 72 h after deactivation of stimulation, we encountered recovery at OFF_72h_ only in a few patients but not across the entire group (Fig. 1 D). Our follow up data after stimulation adjustment with pulse width reduction suggests (Supplementary Fig. S5), that a 72 h wash out phase may not be enough in all patients to recover from rebound ataxia and that an improvement at OFF_72h_ is not a prerequisite for recovery. Generally, our sample of patients differed from that reported by Reich et al. (2016). A longer disease duration and time elapsed since DBS implantation (and assumably duration of therapy escape) in our study population may have led to the different result. Furthermore, Reich et al. (2016) excluded patients with neuropathy, which may have had an impact on both evolution of the delayed therapy escape and recovery over 72 h of paused stimulation. However, in our patients the values of pallesthesia showed possible correlations with signs of ataxia only at OFF_0h_ and OFF_72h_ but neither at ON nor with their development over 72 h of paused stimulation (Supplementary Fig. S4). So proprioceptive input seems to be a relevant factor to cope with the sudden disequilibrium of the rebound situation, but not for the ON state.

Our exploratory analyses found that out of all directly accessible tremor features only the rebound (OFF_0h_) frequency of postural tremor showed correlations of large effect size with signs of ataxia at ON, with low frequencies signaling more ataxia. In general these findings align with computational models of essential tremor suggesting frequency increases with thalamic stimulation, while a decrease of frequency occurs with up-regulation of GABA receptors in the dentate nucleus (Zhang and Santaniello, 2019), which may represent the neuroplastic process leading to the delayed therapy escape.

In all but one patient ataxia and tremor were more pronounced on the non-dominant side. The single patient affected more on the dominant side differs from the rest of the patients due to the earliest onset of therapy escape amongst the group (13 months postoperatively). A possible explanation includes varying compensatory mechanisms resulting in different durations of the therapeutic benefit of DBS.

### 4.2 Delayed therapy escape: A long-term chronic DBS syndrome?

**Fig. 6:**
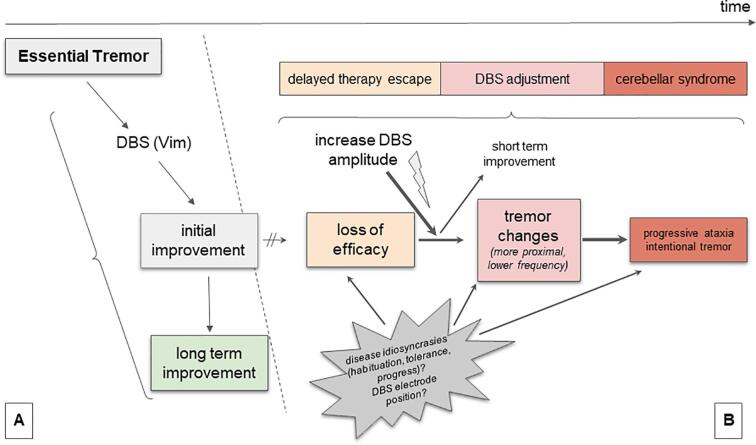
Proposed role of stimulation induced ataxia in delayed therapy escape (B) compared to patients without therapy escape (A).

Delayed therapy escape has been hypothesized to be a correlate of an undesirable compensatory neuroplastic process affecting cerebellar circuits (Fasano and Helmich, 2019, Reich et al., 2016) - a concept that would explain some similarities but also differences between immediate stimulation-induced ataxic features and delayed therapy escape. We propose based on our new findings that ataxia plays a major role in delayed therapy escape (Fig. 6). In our interpretation the disruption of neural transmission by thalamic stimulation (DBS) causes an adaptive equilibrium producing mild cerebellar symptoms in all patients with essential tremor and thalamic stimulation to a certain degree, which can mostly be compensated over a long time (Fig. 6A). Patients with a delayed therapy escape, however, gradually develop a fragile equilibrium with slowly progressive and phenotypically altered tremor and ataxia over months and years (Fig. 6B). Therapy escape could thus be interpreted as a phenomenon where ET is replaced with stimulation induced cerebellar tremor over time (and typically under increased DBS amplitudes). The observed tremor often changes its features under stimulation to a more cerebellar (atactic) tremor (intentional, more proximal, lower frequency) which is potentially an indicator for the gradually increasing functional lesion of the cerebellar projection with a silencing of the synaptic transmission in the thalamus through high frequency stimulation (DBS). A sudden stimulation switch OFF exacerbates ataxic symptoms unmasking the underlying disequilibrium. One might speculate that at the same time the switch off might re-open the synaptic transmission in the thalamus and lead to chaotic information transfer to the motor cortex out of a now - and stimulation induced - highly active cerebellum. Therefore stimulation induced ataxia (functional lesion) is potentially different from ataxia after stimulation cessation (transmission of chaotic signals out of cerebellum) and also a reason why patients often vote for a re-introduction of stimulation (functional lesion) after stimulation cessation with increased ataxia. This might also be the reason why some authors in such situations opt for a thalamic lesioning approach (Fasano and Helmich, 2019). The reasons for this disequilibrium are presumably related to disease idiosyncrasies (tolerance, habituation, progress) and/or DBS electrode position and - speculatively - the mere presence of DBS itself with a coincidence of partial synaptic silencing in the Vim and antidromic dentate over-activation as described above (Fig. 6).

This whole line of argumentation and the suspected high incidence of detrimental effects of continuous stimulation point to the necessity to develop advanced stimulation strategies (like closed loop DBS) which potentially can circumvent these effects while at the same time granting high efficacy (Cernera et al., 2021).

### 4.3 Electrode positions

Mean effective electrode positions and positions of contacts penetrating the thalamic (MCP) plane along the leads were as expected within the typical range. We were not able to draw further conclusions out of the mere ACPC based or MNI positions.

### 4.4 Metabolic activity in patients with therapy escape

Our analyses of PET imaging data demonstrates that bilateral thalamic stimulation in delayed therapy escapers leads to a pattern with bilaterally increased metabolic activity in the thalamus (extending into the midbrain) and dentate nuclei, which has not been reported before. ROI-based analysis of the thalamus showed that average thalamic metabolism of controls ranges between Stim ON and OFF condition of patients (Fig. 4), explaining less pronounced group differences between patient conditions and the control group (despite large effect sizes of between stimulation differences). Whether the relative thalamic hypometabolism we observed in Stim OFF condition in comparison to healthy controls is caused by insufficient recovery after 3 days stimulation holiday, loss of neuronal function caused by chronic stimulation, or disease progression needs further exploration.

A possible explanation for the increased metabolism in the dentate nucleus is an antidromic activation of cerebellar structures via the fasciculus cerebellothalamicus (dentatothalamic fibers), which is an observation different to the results of Reich et al. (2016). They had found extrathalamic activation in the cerebellar nodule only and assumed current spread into the adjacent ascendant uncinate tract. Antidromic stimulation of the dentate nucleus may be an idiosyncratic feature of thalamic stimulation for tremor to a certain degree. We cannot elaborate on this speculation further, because we did not conduct PET in patients without therapy escape and Reich et al. (2016) did not report findings from this comparison either. In addition to the methodological differences to the study of Reich et al. (2016) discussed above, the technical factors like different scanners and reconstruction protocols may explain diverging findings. In contrast to earlier studies, patients in this study underwent PET on a novel fully-digital PET/CT scanner that provides superior spatial resolution in comparison to conventional clinical scanners, allowing for quantitative imaging of small brain structures like brainstem and cerebellar nuclei (Speck et al., 2020). Earlier studies showed no increase in cerebellar glucose metabolism in patients with essential tremor without DBS compared to healthy controls (Hallett and Dubinsky, 1993, Song et al., 2015). Examining regional cerebral blood flow (rCBF) instead of regional cerebral glucose metabolism Perlmutter et al. (2002) found signs of increased neuronal activity at the thalamic stimulation site, but not in the cerebellum. This could be due to comparing instances of ON and OFF_0h_ in patients with essential tremor and unilateral left Vim-DBS but not necessarily patients with therapy escape. Further PET results are lacking in patients with essential tremor and thalamic DBS with or without therapy escape. PET studies on thalamic DBS in parkinsonian tremor found decreased rCBF predominantly in medial cerebellar regions associated with stimulation and considered this to be a result of antidromic stimulation of cerebellothalamic projections (Davis et al., 1997, Deiber et al., 1993). Although obtained in patients with Parkinson’s disease, these results reflect metabolic patterns of effective thalamic stimulation in the early course after DBS implantation, which therefore may relate to essential tremor without stimulation-induced cerebellar syndrome. But comparisons have to be interpreted with care as substantial methodological differences apply.

In addition to the signs of antidromic stimulation of the dentate nucleus, higher metabolic activity in the ipsilateral and contralateral thalamus at Stim ON possibly relates to lower frequencies of postural rebound tremor at OFF_0h_ on the most affected side (Supplementary Fig. S6). Thus, a higher metabolic activity at the stimulation site could be suggestive of therapy escape or chronic overstimulation. Because exploratory analyses did not find associations with signs of ataxia or total electrical energy delivered (TEED) by DBS, we propose that additional factors play a role in the evolution of therapy escape, in particular addressing decussating and non-decussating portions of the DRT by the stimulation.

## 5 Limitations

At this time there is no commonly accepted definition for therapy escape. Here we generally adopted the proposed definition by Fasano and Helmich (2019). However, due to the retrospective nature of the analysis including patients who underwent implantation at another institution, some of the information classifying the patients as therapy escapers had to be gathered from medical records and patients’ reports and are not based on parametric or operationalized testing. Nevertheless the time of subjectively perceived satisfaction with DBS treatment suggests that the proposed definition of Fasano and Helmich is met by all patients in this study.

This work suffers from the typical problems of a retrospective data collection with missing data. The small sample size is an additional limitation to this study. As a consequence of this, no corrections for multiple comparisons were applied and only exploratory analyses were performed (except voxel-wise analysis of PET data).

Moreover, this study shows strong indication of antidromic stimulation of the dentate nucleus in addition to thalamic stimulation during Stim ON condition. Despite a large effect of this observation, no link to clinical data was found in this cohort possibly due to already discussed limited sample size and missing clinical ratings in some of the patients. To confirm the findings and verify the discussed mechanism a fully powered trial for the respective hypothesis will be necessary. PET data per se need to be interpreted with caution since there is no Vim DBS patient cohort which contrasts our findings. As a consequence we cannot be sure if the overactive dentate nucleus is not a common phenomenon under stimulation, which we find unlikely based on clinical grounds.

A missing control group without therapy escape limits the generalisability of our results as exclusive effects of delayed therapy escape. However, supplemental follow up data underlines the role of rebound tremor frequency.

Another limitation is the short walking distance and turn involved in the timed-up-and-go task examined with the motion capture, impeding a reliable calculation of variability measures of gait parameters (Kroneberg et al., 2018), which would be desirable for analysis of gait ataxia (e.g. coefficient of variation of step length). At the same time, step length can already be reliably assessed with a few walking cycles (other than its coefficient of variation) (Kroneberg et al., 2018) and has been shown to increase significantly in the further course of degenerative cerebellar ataxias (Serrao et al., 2017) and can also differ significantly between patients and healthy controls (Buckley et al., 2018, Palliyath et al., 1998).

## 6 Conclusion

There is an ongoing scientific debate on the mechanisms involved in delayed therapy escape after Vim DBS mostly focusing on disease progression versus habituation (Fasano and Helmich, 2019, Favilla et al., 2012, Peters and Tisch, 2021). We here add a further possibility, namely a direct effect of long term DBS. As such our data adds to the body of evidence of delayed therapy escape, but further research is needed to disentangle the different aspects of this complex phenomenon. Moreover, our data strengthens the case for the development of closed loop DBS approaches which in the future might help to circumvent side effects of long term and chronic stimulation (Opri et al., 2020).

Our results suggest that rebound frequency of postural tremor upon switching off thalamic stimulation for essential tremor can unmask an underlying therapy escape through a delayed stimulation-induced cerebellar syndrome. As a consequence, it might be desirable to monitor rebound tremor frequencies in patients with thalamic DBS for essential tremor systematically over time. Furthermore, we provide evidence that delayed therapy escape may be associated with increased metabolic activity in the thalamus and dentate nucleus.

## CRediT authorship contribution statement

**Bastian E.A. Sajonz:** Conceptualization, Methodology, Validation, Formal analysis, Investigation, Resources, Data curation, Writing – original draft, Visualization. **Marvin L. Frommer:** Conceptualization, Software, Formal analysis, Investigation, Writing – review & editing. **Isabelle D. Walz:** Formal analysis, Investigation, Writing – review & editing. **Marco Reisert:** Software, Writing – review & editing. **Christoph Maurer:** Resources, Writing – review & editing. **Michel Rijntjes:** Resources, Writing – review & editing. **Tobias Piroth:** Software, Writing – review & editing. **Nils Schröter:** Writing – review & editing. **Carolin Jenkner:** Formal analysis, Writing – review & editing. **Peter C. Reinacher:** Writing – review & editing. **Joachim Brumberg:** Writing – review & editing. **Philipp T. Meyer:** Supervision, Resources, Writing – review & editing. **Ganna Blazhenets:** Conceptualization, Methodology, Software, Validation, Formal analysis, Data curation, Writing – original draft, Visualization. **Volker A. Coenen:** Conceptualization, Supervision, Writing – review & editing.

## Declaration of Competing Interest

The authors declare the following financial interests/personal relationships which may be considered as potential competing interests: BEAS receives a research grant from Ceregate (Hamburg, Germany) unrelated to this publication. NS received a grant from the Berta-Ottenstein-Programme for Clinician Scientists, Faculty of Medicine,University of Freiburg, and received payment for a lecture sponsored by Abbvie. PCR receives research support from: Else Kröner-Fresenius Foundation (Germany) and Fraunhofer Foundation (Germany). He is a consultant for Boston Scientific (USA), Inomed (Germany) and Brainlab (Germany). JB received a grant from the German Research Foundation (Deutsche Forschungsgemeinschaft, DFG). PTM received honoraria from GE (presentation, consultancy) and Philips (presentation). VAC receives a collaborative grant from BrainLab (Munich, Germany). He is a consultant for Ceregate (Hamburg, Germany), Cortec (Freiburg, Germany) and Inbrain (Barcelona, Spain). He has ongoing IITs with Medtronic (USA) and Boston Scientific (USA). GB, IW, CM, MLF, MR, TP, have nothing to report. All declared interests are outside of the submitted work.

## Data Availability

Data will be made available on request.
