# Supplementary material for: Unravelling delayed therapy escape after thalamic deep brain stimulation for essential tremor? – Additional clinical and neuroimaging evidence
Source: Neuroimage Clin. 2022 Aug 11;36:103150. doi: 10.1016/j.nicl.2022.103150 (PMC9402391; doi:10.1016/j.nicl.2022.103150)
Supplement: Supplementary data 1 [file mmc1.docx]

**Supplementary Material**


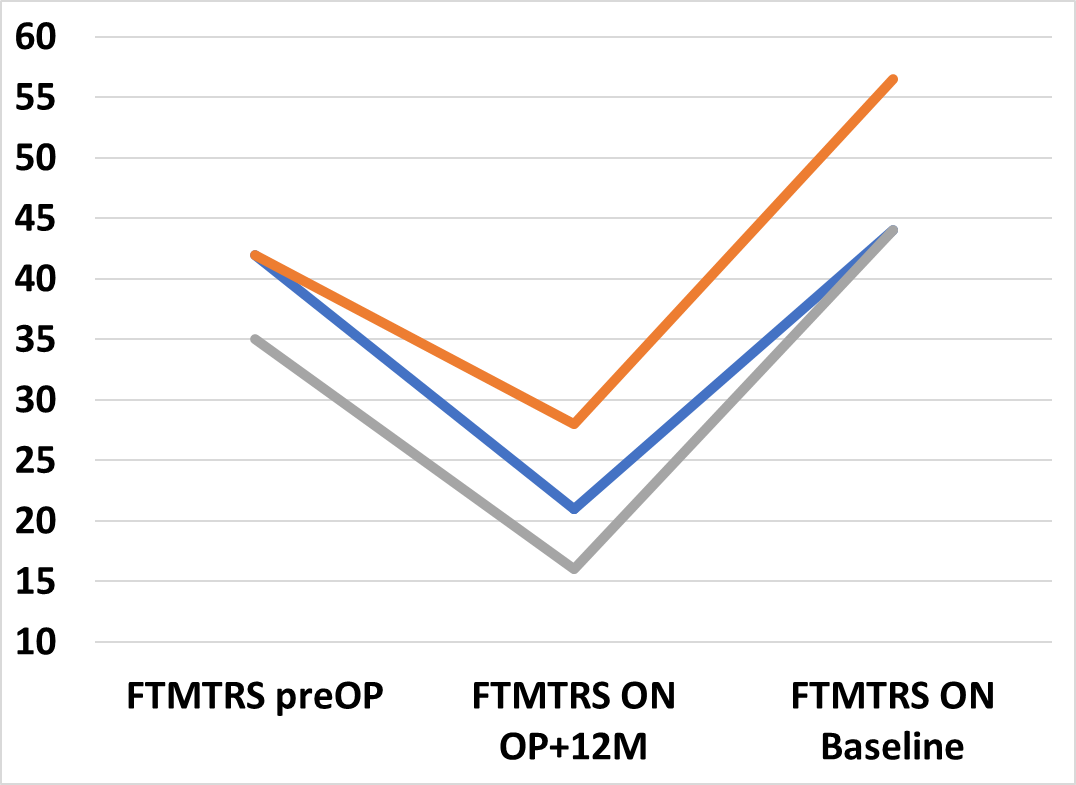


**Figure S1: Development of FTMTRS preceding baseline of this study in the 3 patients with available values from preOP and 12 months postOP.**

__________________________________________________


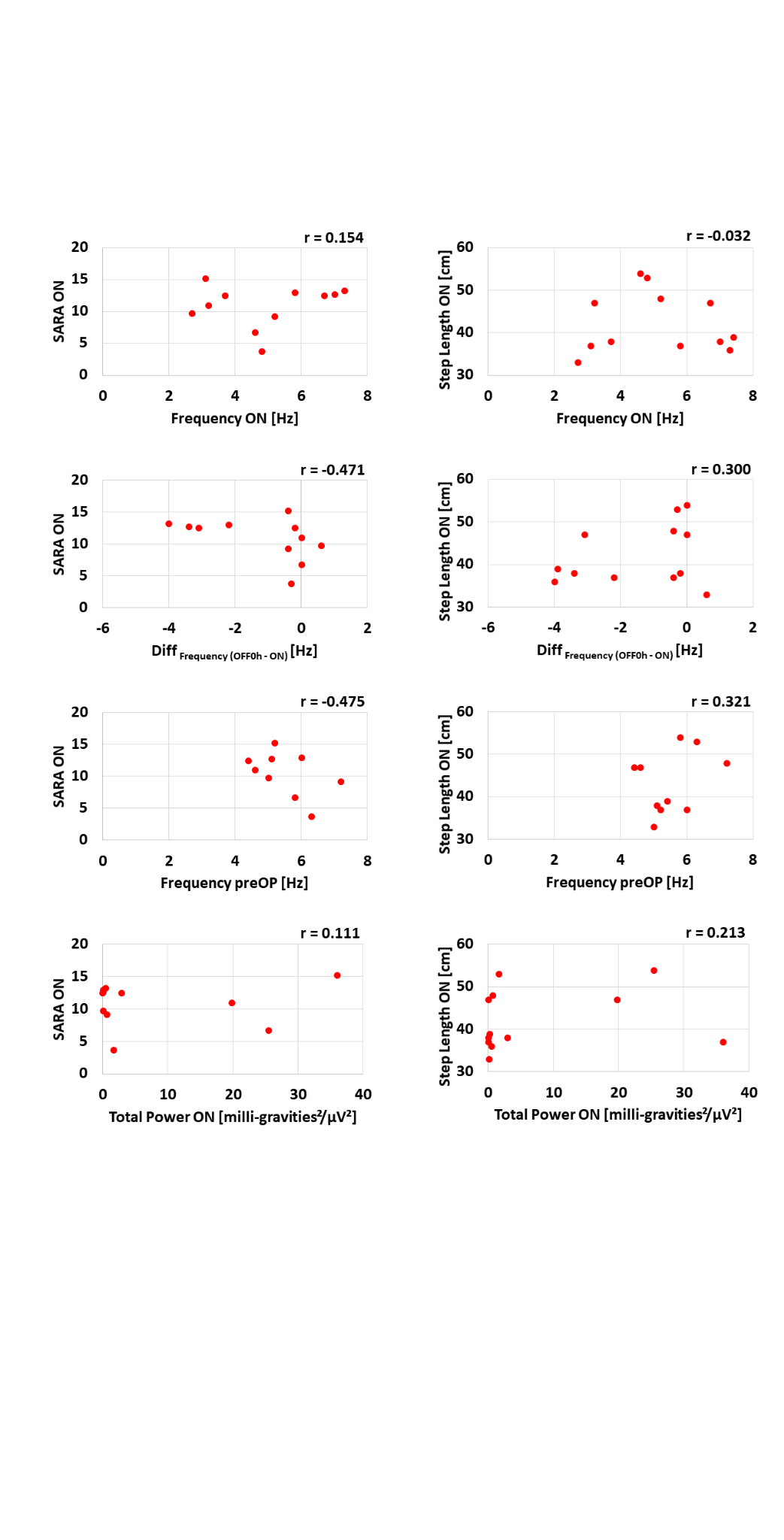
**Figure S2: Further correlation analyses in search of a directly accessible marker of postural tremor of the most affected side.** From top to bottom: frequency of postural tremor at ON, difference of frequency of postural tremor OFF_0h_ - ON, frequency of postural tremor preOP, total power of postural tremor at ON) for signs of ataxia (SARA and step length) did not show any correlation with large effect size. If item 6 (nose-finger test measuring tremor) of the SARA is removed from analysis, all correlations remain without relevant change in the level of their Pearson’s product-moment correlation coefficient. Missing values for SARA in 1 patient and preoperative tremor frequency in 2 patients.

__________________________________________________________________________________________


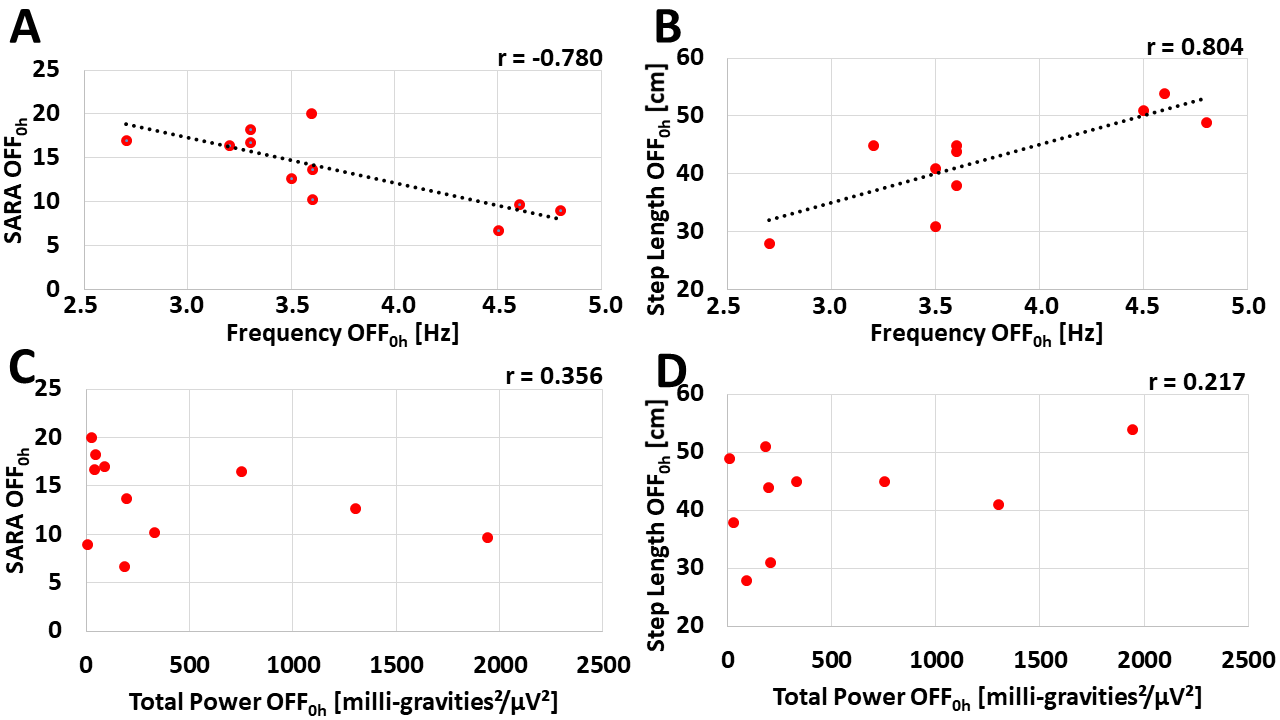


**Figure S3: Scatter plots depicting the association between the tremor related measures (frequency and total power) and signs of ataxia (SARA and step length) at OFF_0h_.** Correlation of (A) SARA OFF_0h_ with frequency OFF_0h_, (B) step length at OFF_0h_ with frequency OFF_0h_, (C) SARA OFF_0h_ with total power OFF_0h_ and (D) step length OFF_0h_ with total power OFF_0h_. Missing values for SARA in 1 patient. Missing values for step length at OFF0h in 2 patients. Regression lines are depicted for possible correlations (i.e. exceeding |r| ≥ 0.5). **__________________________________________________________________________________________**

**
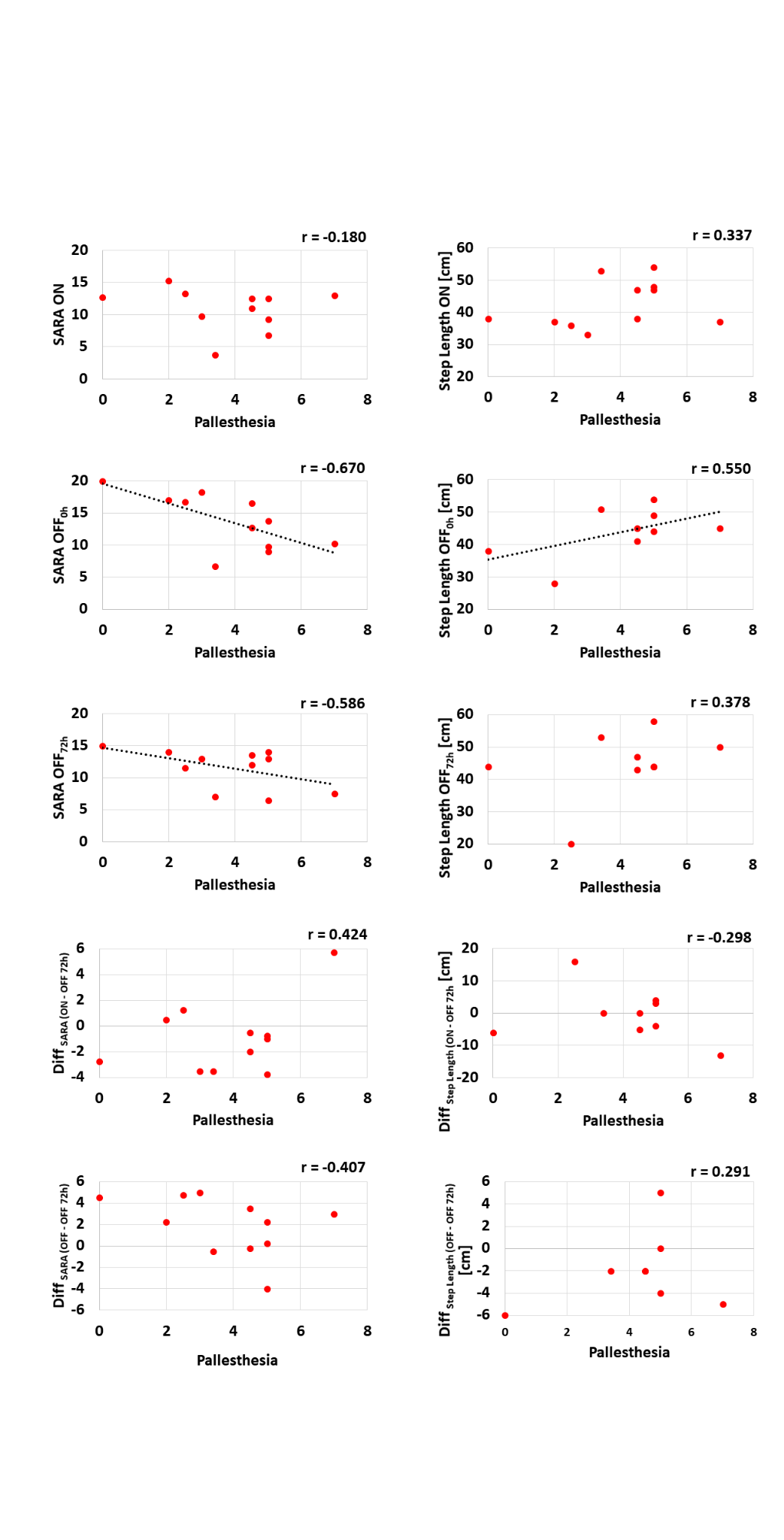
**

**Figure S4: Correlation analyses to demonstrate the impact of malleolar pallesthesia (mean of both sides) on signs of ataxia (left: SARA, right: step length) at different time points and their development over time.** From top to bottom: ON, OFF_0h_, OFF_72h_, Difference ON - OFF_72h_, Difference OFF_0h_ - OFF_72h_. Regression lines are depicted for possible correlations (i.e. exceeding |r| ≥ 0.5). Missing values: pallesthesia and SARA in one patient; step length missing for one patient both in the OFF_0h_ and OFF_72h_ condition and two other patients in either the OFF_0h_ or OFF_72h_ condition. As a result two values are missing for the difference in step length ON - OFF72h and three values for the difference OFF_0h_ - OFF_72h_. Coincidentally, two patients have the identical combination of values for pallesthesia and step length at OFF_72h_ and another two patients for pallesthesia and the difference of step length OFF_0h_ - OFF_72h_. Consequently, these cases are represented by one dot in the corresponding scatterplot.

__________________________________________________________________________________________


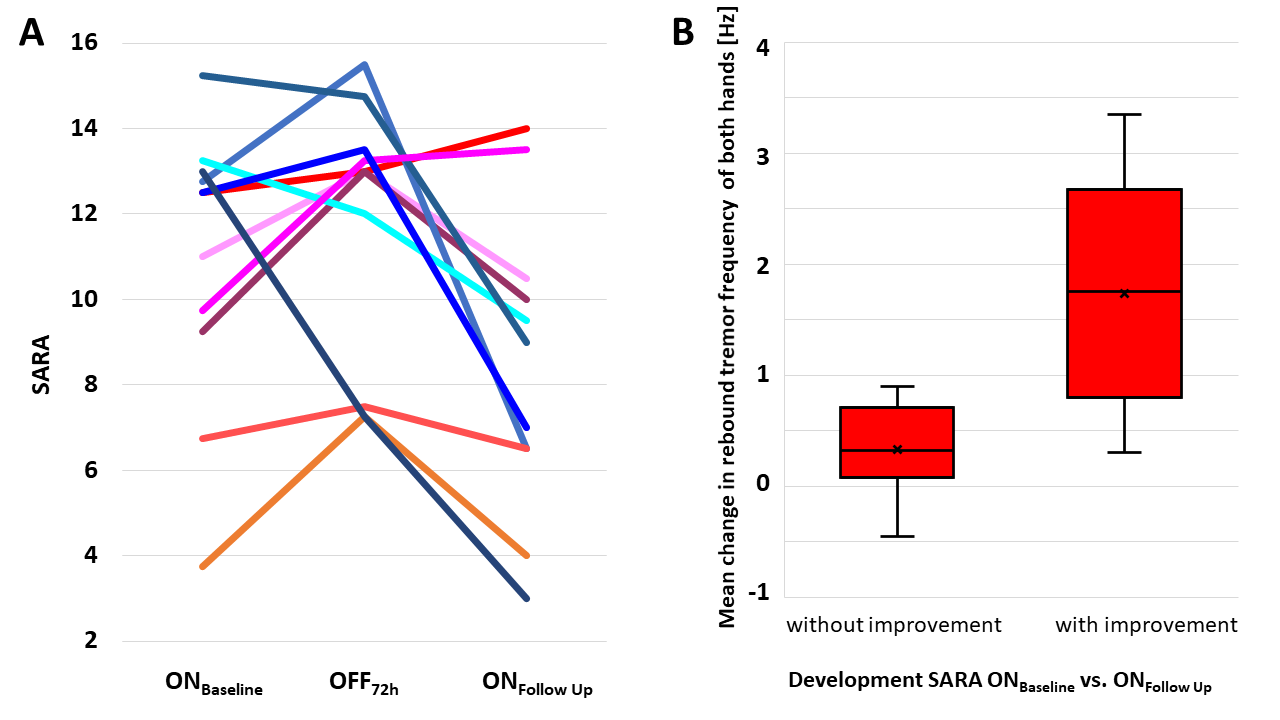


**Figure S5 (to be printed in color): Illustration of the further course based on follow up data obtained 24 ± 10 months (mean ± SD) after OFF_72h_.** (A) Development of individual SARA values. Patients with meaningful improvement (> 1.5 points) are depicted in blueish colors and patients without improvement in redish colors. Patients with improvement between baseline and follow up did not necessarily improve with 72 hours of paused stimulation. (B) Boxplots illustrating the mean change of both hand’s rebound (OFF_0h_) tremor frequency (i.e. positive values indicate and increase from baseline to follow up) in patients with and without meaningful improvement according to SARA at follow up (Cohen’s d = 0.843).

__________________________________________________________________________________________

**
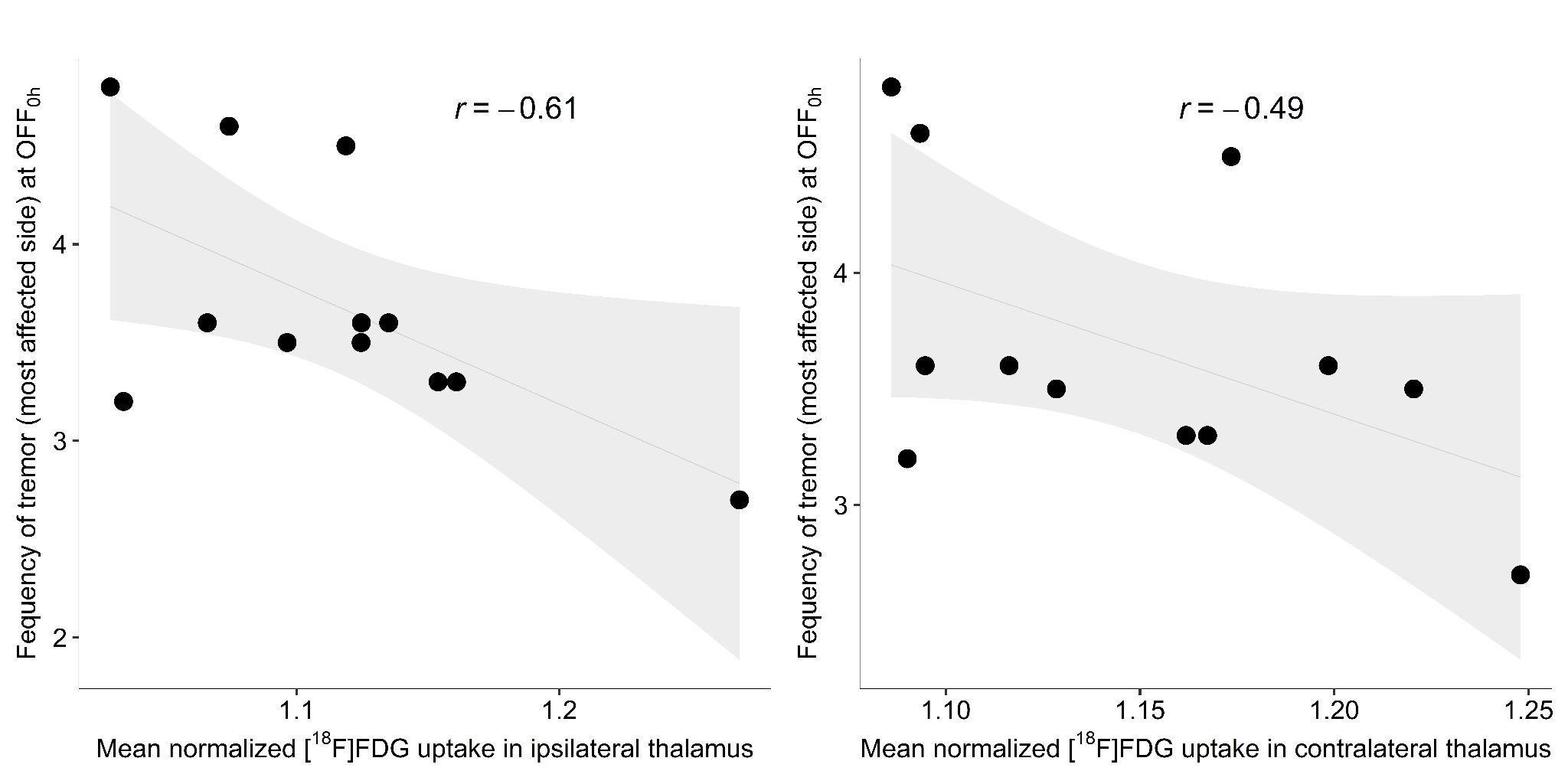
**

**Figure S6: Scatterplots showing the association between rebound frequency of postural tremor (OFF_0h_) and mean normalized [^18^F]FDG-uptake in the thalamus at ON.** Patients with a higher [^18^F]FDG-uptake in the thalamus at ON tend to have lower frequencies of rebound tremor upon OFF_0h_

**__________________________________________________________________________________________**

**Table S1:** ACPC coordinates of active (cathodal) electrode contacts in mm (additionally % for y) with regard to PC

|  | **Left** | | | | **Right** | | | |
| --- | --- | --- | --- | --- | --- | --- | --- | --- |
| **#** | **x (lat) [mm]** | **y (ant) [mm]** | **y(ant) [%]** | **z (inf)**  **[mm]** | **x (lat)**  **[mm]** | **y (ant)**  **[mm]** | **y (ant)**  **[%]** | **z (inf)**  **[mm\|** |
| **1** | **11.0** | **4.3** | **17.4** | **1.4** | **10.9** | **5.1** | **20.6** | **4.4** |
| **2** | **10.8** | **5.7** | **22.4** | **2.4** | **10.2** | **5.3** | **20.8** | **1.9** |
| **3** | **12.6** | **6.6** | **26.7** | **-3.0** | **13.2** | **7.6** | **30.5** | **-1.5** |
| **4** | **15.0** | **8.3** | **30.6** | **-1.2** | **13.3** | **6.1** | **22.8** | **0.0** |
| **5** | **13.7** | **7.9** | **30.2** | **-4.2** | **14.1** | **8.0** | **30.7** | **-3.8** |
| **6** | **15.9** | **9.9** | **38.9** | **1.6** | **13.2** | **9.0** | **35.3** | **0.5** |
| **7** | **11.4** | **7.9** | **29.4** | **2.1** | **13.2** | **8.6** | **31.9** | **0.4** |
| **8** | **14.2** | **8.0** | **32.9** | **2.2** | **14.4** | **7.1** | **29.2** | **2.5** |
| **9** | **10.6** | **8.7** | **32.1** | **2.9** | **8.6** | **6.5** | **23.9** | **1.4** |
| **10** | **12.2** | **3.8** | **16.2** | **1.4** | **10.1** | **2.6** | **10.8** | **1.7** |
| **11** | **8.74** | **5.3** | **22.0** | **3.2** | **10.1** | **5.9** | **24.6** | **1.2** |
| **12** | **12.7** | **5.8** | **21.9** | **1.1** | **15.9** | **8.8** | **32.9** | **-3.1** |
| **Mean**  **±SD** | **12.4**  **± 2.1** | **6.9**  **± 1.9** | **26.7**  **± 6.8** | **0.8**  **± 2.4** | **12.3**  **± 2.2** | **6.7**  **± 1.9** | **26.1**  **± 6.8** | **0.5**  **± 2.3** |

lat, lateral; ant, anterior; y%, y given as ratio y/ACPC distance, inf, inferior; in cases with more than one active (cathodic) contact (including interleaved programs) value of the resulting center along the electrode is displayed; inf, inferior to ACPC plane

**Table S2:** ACPC coordinates in mm (additionally % for y) of penetration of electrode leads with ACPC-plane (z = 0) with regard to PC

|  | **Left** | | | **Right** | | |
| --- | --- | --- | --- | --- | --- | --- |
| **#** | **x (lat)**  **[mm]** | **y (ant)**  **[mm]** | **y (ant)**  **[%]** | **x (lat)**  **[mm]** | **y (ant)**  **[mm]** | **y (ant)**  **[%]** |
| **1** | **11.6** | **4.8** | **19.4** | **13.1** | **6.3** | **25.6** |
| **2** | **11.2** | **6.4** | **25.1** | **11.4** | **6.0** | **23.4** |
| **3** | **11.1** | **4.9** | **19.8** | **12.4** | **6.4** | **25.7** |
| **4** | **14.5** | **7.6** | **28.2** | **13.3** | **6.1** | **22.8** |
| **5** | **11.7** | **6.1** | **23.4** | **12.1** | **6.2** | **23.8** |
| **6** | **16.7** | **10.8** | **42.6** | **13.3** | **9.2** | **36.2** |
| **7** | **12.5** | **9.8** | **36.4** | **13.5** | **8.9** | **33.0** |
| **8** | **15.9** | **11.0** | **45.2** | **15.9** | **9.4** | **38.9** |
| **9** | **12.8** | **11.3** | **41.3** | **9.2** | **7.5** | **27.4** |
| **10** | **12.9** | **4.6** | **19.6** | **10.9** | **3.6** | **15.4** |
| **11** | **10.5** | **7.3** | **30.7** | **11.6** | **6.7** | **28.1** |
| **12** | **13.2** | **6.3** | **23.7** | **14.3** | **6.8** | **25.4** |
| **Mean**  **± SD** | **12.9**  **± 1.9** | **7.6**  **± 2.5** | **29.6**  **± 9.5** | **12.6**  **± 1.7** | **6.9**  **± 1.6** | **27.1**  **± 6.4** |

lat, lateral; ant, anterior; y %, y given as ratio y/ACPC distance

**Table S3:** Stimulation parameters

| **#** | **electrode lead type** | **(L)**  **contacts** | **(L)**  **current**  **[mA]** | **(L)**  **pulsewidth [µs]** | **(R)**  **contacts** | **(R)**  **current**  **[mA]** | **(R)**  **pulsewidth**  **[µs]** | **(R+L)**  **Frequency**  **[Hz]** |
| --- | --- | --- | --- | --- | --- | --- | --- | --- |
| **1** | **3389** | **1: C+,3-**  **2: C+, 2-** | **1: 2.8**  **2: 2.7** | **60** | **1: 4+, 3-**  **2: C+, 2-** | **1: 1.4**  **2: 2.4** | **60** | **125** |
| **2** | **3389** | **1+, 2-, 3-** | **3.8** | **30** | **1+, 2-, 3-** | **4.9** | **50** | **180** |
| **3** | **3387** | **C+, 2-** | **2.8** | **60** | **C+, 2-** | **3.1** | **60** | **160** |
| **4** | **3389** | **1+, 2-** | **5.4** | **90** | **3+, 1-, 2-** | **5.2** | **90** | **180** |
| **5** | **3389** | **2+, 3-** | **6.0** | **30** | **1+, 2-, 3- 4-** | **7.4** | **30** | **180** |
| **6** | **3387** | **3+, 1-, 2-** | **4.5** | **150** | **3+, 1-, 2-** | **6.5** | **150** | **180** |
| **7** | **3389** | **C+, 1-** | **2.9** | **90** | **C+, 1-, 2-** | **5.5** | **90** | **130** |
| **8** | **3389** | **1: C+, 2-, 3-**  **2: C+, 1-** | **1: 4.4**  **2: 0.9** | **60** | **C+, 1-, 2-** | **4.4** | **60** | **125** |
| **9** | **3389** | **C+, 3-, 4-** | **3.5** | **60** | **C+, 3-, 4-** | **3.5** | **60** | **110** |
| **10** | **3389** | **1+, 2-, 3-** | **4.1** | **60** | **C+, 1-** | **2.2** | **60** | **180** |
| **11** | **3389** | **C+, 2-** | **2.7** | **90** | **C+, 2-** | **2.8** | **90** | **180** |
| **12** | **Cartesia** | **1+, 2(a-c) - (25 % each),**  **3(a-c) - (8 % each)** | **8.5** | **50** | **2(a-c)+ (33 % each),**  **3(a-c)- (5 % each),**  **4- (85 %)** | **4.5** | **50** | **149** |
| **Mean ± SD** | **n/a** | **n/a** | **3.9**  **± 1.8** | **69**  **± 33** | **n/a** | **4.1**  **± 1.8** | **71**  **± 31** | **157**  **± 27** |

n/a, not applicable; R, right; L, left; Model 3389 and 3387 electrodes from Medtronic (interspacing 0.5 and 1.5 mm, respectively); Cartesia directional electrode from Boston-Scientific; two values given in patients with interleaved programs; Logic of contact naming, irrespective of model: inferior most contact “1”, superior most contact “4”. Cartesia electrode segmented rings are named “2 a-c” and “3 a-c” (instead of 2-4 and 5-7, respectively). C, case of INS (indicating monopolar stimulation mode).
